# Supplementary material for: Multiple exposures, reinfection and risk of progression to active tuberculosis
Source: R Soc Open Sci. 2019 Mar 20;6(3):180999. doi: 10.1098/rsos.180999 (PMC6458392; doi:10.1098/rsos.180999)

Supplemental Information: Multiple Exposures, Reinfection, and Risk of Progression to Active Tuberculosis

Reinfection Model

In this model, each infection confers a constant risk of resulting in active disease. However, not all exposures become infections. We define the following random variables: C is a binary random variable representing whether or not an individual becomes a case; I is the number of infections; and E is the number of exposures.

Parameters

- p: probability of becoming an infectious case per infection
- ζ: probability an exposure becomes an infection

Probability of becoming a case with i infections:

$$\Pr[C = 1|I = i] = 1 - (1 - p)^i \tag{1}$$

Probability of i infections given e exposures, where $e > i \geq 0$.

$$\Pr[I = i|E = e] = \binom{e}{i}(1 - \zeta)^{e-i}\zeta^i \tag{2}$$

Note, everyone in the dataset has at least one infection. Thus, the probability that an individual k with e_k exposures becomes a case is given by:

$$x_k(p, \zeta) = 1 - (1 - p) - \sum_{i=0}^{e_k}(1 - p)^i \binom{e_k - 1}{i}(1 - \zeta)^{e_k-i-1}\zeta^i \tag{3}$$

The likelihood is given by:

$$\mathcal{L}(p, \zeta) = \sum_k \delta_{1c_k} x_k(p, \zeta) + (1 - \delta_{1c_k})(1 - x_k(p, \zeta)) \tag{4}$$

where δ_{1c_k} is the Kronnecker delta function, which takes on the value of one if the kth individual became a case ($c_k = 1$), and is zero otherwise.

Parameter Estimates and Confidence Intervals

Parameter	Estimate	95% Confidence Interval
p	0.117	(0.0604,0.213)
ζ	0.179	(0.0549,0.452)

Threshold Model

In this model, the risk of disease scales with exposures. We use the error function to model the increase in risk of infection with increasing exposures. The error function is defined as follows:

$$\mathrm{erf}(x) = \frac{2}{\sqrt{\pi}} \int_0^x \exp(-t^2) dt \tag{5}$$

Let $f(e)$ be the risk of becoming a case as a function of the number of exposures e.

$$f(e) = h \ (\mathrm{erf}(s(e - e_c) + 1) - h \ (\mathrm{erf}(s(1 - e_c) + 1) + z \tag{6}$$

The parameters are as follows:

- h: parameterizes how large the jump is between risk for low and high exposures. Note the risk difference is twice this parameter.
- s: parameterizes how quickly the risk increases between its value for low exposures and high exposures.
- e_c: location where risk is at its midpoint value between the risk for low and high exposures.
- z: parameterizes baseline risk (one exposure).

Parameter Estimates and Confidence Intervals

Parameter	Estimate	95% Confidence Interval
h	0.226	(0.136,0.375)
s	0.291	(0.049,1.73)
e_c	17.7	(13.9,22.5)
z	0.148	(0.0927,0.236)

Increasing Risk Model–Nested within Threshold Model

The inflection point is constrained to be at zero. $f(e)$, the risk of becoming a case as a function of the number of exposures e, takes on the following form.

$$f(e) = h \text{ erf}(s \cdot e) - h \text{ erf}(s) + z \tag{7}$$

The parameters are as follows:

- h: parameterizes how large the jump is between risk for low and high exposures.
- s: parameterizes how quickly the risk increases between its value for low exposures and high exposures.
- z: parameterizes baseline risk (one exposure).

Parameter Estimates and Confidence Intervals

Parameter	Estimate	95% Confidence Interval
h	2.33	(0.00137,3940)
s	0.00611	(3.39×10^{-6},11)
z	0.118	(0.0639,0.216)

Simulation

To determine the dynamic impact of reinfection within a year of initial infection, we performed simulations from modified Reed-Frost models for two years following an initial influx of cases. We parameterized these models with the maximum likelihood estimates from the reinfection and threshold models. In the main text, we presented results for generating a number of discrete exposure events for each uninfected individual from the negative binomial distribution with mean one-tenth times the number of infectious individuals and a dispersion parameter of one-tenth. These exposures were then randomly assigned to each of the cases. The total number of unique cases which each individual was exposed to were tallied, giving a number of unique cases each individual was exposed to, comparable to the number of exposures as collected for the dataset. Alternative parameterizations are given here. For these parameterizations, we compare the threshold and reinfection models to a constant risk model parameterized from the reinfection model. While alternative parameterizations can affect the expected outbreak sizes, the reinfection and threshold models always produce significantly larger outbreak sizes than the constant-risk model for large numbers of initial exposures.

Scenario 1: Larger Mean Number of Infections per Case

We generated a number of discrete exposure events for each uninfected individual from the negative binomial distribution with mean two-tenths times the number of infectious individuals and a dispersion parameter of one-half.

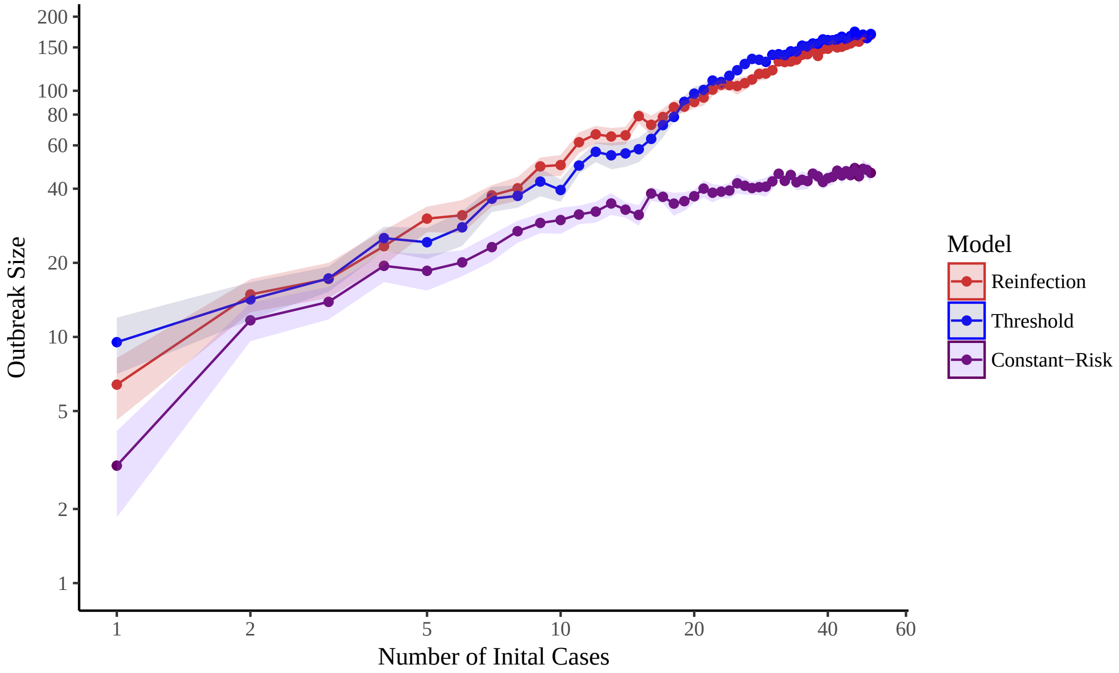

Scenario 2: Less Dispersion in Generation of Discrete Exposure Events

We generated a number of discrete exposure events for each uninfected individual from the negative binomial distribution with mean one-tenth times the number of infectious individuals and a dispersion parameter of 1.

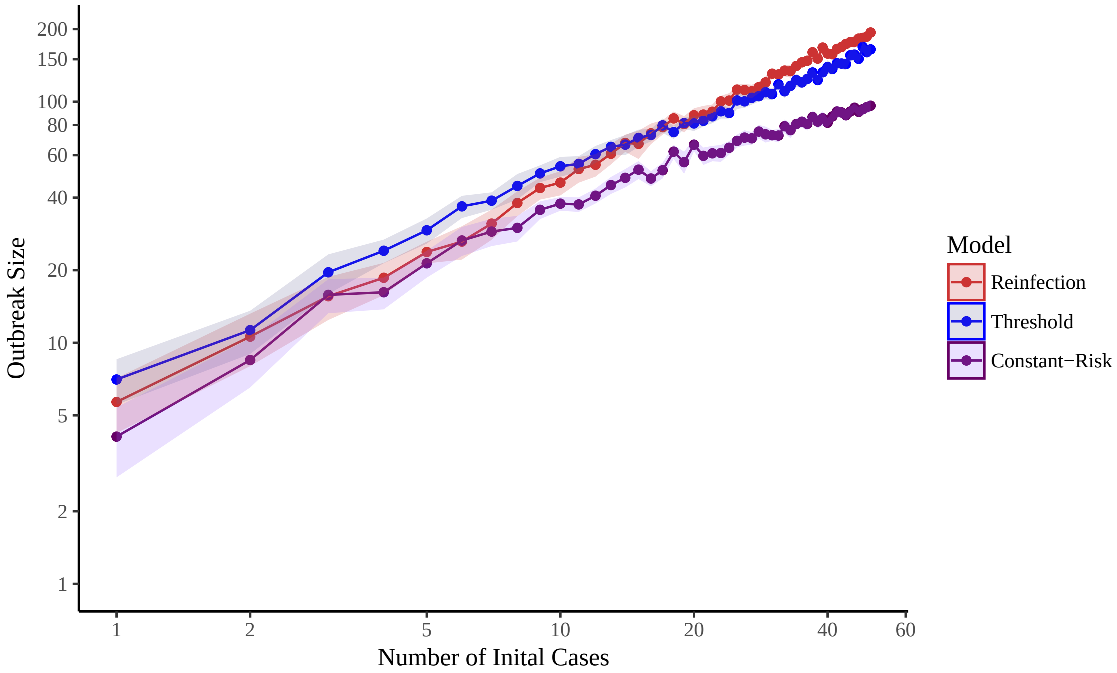

Supplement: Supplemental Information [file rsos180999supp1.pdf]
